# Neuroplastin in Neuropsychiatric Diseases

**DOI:** 10.3390/genes12101507

**Published:** 2021-09-26

**Authors:** Xiao Lin, Yi Liang, Rodrigo Herrera-Molina, Dirk Montag

**Affiliations:** 1 Neurogenetics Laboratory, Leibniz Institute for Neurobiology, Brenneckestr. 6, D-39118 Magdeburg, Germany; xiao.lin@lin-magdeburg.de (X.L.); yi.liang@lin-magdeburg.de (Y.L.); 2 Combinatorial NeuroImaging (CNI), Leibniz Institute for Neurobiology, Brenneckestr. 6, D-39118 Magdeburg, Germany; Rodrigo.Herrera-Molina@lin-magdeburg.de; 3 Centro Integrativo de Biología y Química Aplicada, Universidad Bernardo O’Higgins, Santiago 8307993, Chile; 4 Center for Behavioral Brain Sciences (CBBS), D-39106 Magdeburg, Germany

**Keywords:** plasma membrane calcium ATPase, PMCA, autism, schizophrenia, Alzheimer’s disease, calcium homeostasis, synaptopathy, 15q24 microdeletion syndrome

## Abstract

Molecular mechanisms underlying neuropsychiatric and neurodegenerative diseases are insufficiently elucidated. A detailed understanding of these mechanisms may help to further improve medical intervention. Recently, intellectual abilities, creativity, and amnesia have been associated with neuroplastin, a cell recognition glycoprotein of the immunoglobulin superfamily that participates in synapse formation and function and calcium signaling. Data from animal models suggest a role for neuroplastin in pathways affected in neuropsychiatric and neurodegenerative diseases. Neuroplastin loss or disruption of molecular pathways related to neuronal processes has been linked to various neurological diseases, including dementia, schizophrenia, and Alzheimer’s disease. Here, we review the molecular features of the cell recognition molecule neuroplastin, and its binding partners, which are related to neurological processes and involved in learning and memory. The emerging functions of neuroplastin may have implications for the treatment of diseases, particularly those of the nervous system.

## 1. Introduction

The prevalence of mental disorders, including autism spectrum disorder (ASD) and schizophrenia (SZ) [1,2,3], related to neurodevelopmental deficits and neurodegenerative diseases, is predicted to increase in future decades because of a growing and ageing world population. In addition to its severe effects on cognitive and social communication, its economic burden is a major challenge for patients and for economies at an international level [4,5,6,7]. To understand the pathogenesis of neuropsychiatric disorders, it is important to consider that both genetic and environmental factors can act separately or in combination to play crucial roles in these diseases. Based on genome-wide association studies (GWAS) with large groups of patients, potential mechanisms underlying different psychiatric disorders can be elucidated and directly investigated. As GWAS and next generation sequencing (NGS) have rapidly developed recently, many gene loci have been associated with different neuropsychiatric disorders [8]. For instance, numerous genetic variants have been associated with SZ [9,10,11,12,13] and ASD [14,15,16,17]. Furthermore, a significant genetic correlation exists between ASD and SZ [17,18]. In combination with transgenic mouse models targeting the potential risk genes, the association of different psychiatric disorders has been confirmed for explicit genes such as the SHANK genes [19,20,21,22,23]. Furthermore, gene variants leading to synaptic dysfunction play a critical role as causal factors for these psychiatric disorders [24].

The abnormal expression or function of several proteins can affect synaptic transmission and further impair network activities, such as the excitatory and inhibitory balance, contributing to different neuropsychiatric or neurodegenerative diseases [25,26]. Therefore, numerous efforts have focused on exploring interventions with identified pathogenic mechanisms, although many attempts did not achieve the desired clinical outcome [27,28,29,30,31]. As psychiatric disorders frequently exhibit overlapping symptoms, such as the association of cognitive impairment with ASD and SZ [32], it is essential to understand their underlying cellular mechanisms.

**Figure 1:**
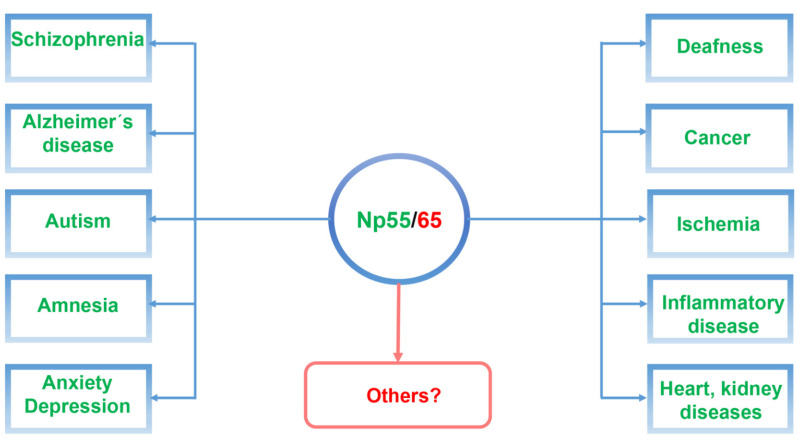
Schematic illustration of neuroplastin Np55/65 as a central component related to neuropsychiatric and neurodegenerative diseases as well as in other diseases associated with neuroplastin malfunctions.

In this review, we will focus on recent studies of the cell recognition molecule neuroplastin (Np) and its gene (*Nptn*/*NPTN*) in relation to psychiatric and neurodegenerative diseases (Figure 1). We propose that targeting neuroplastin may make it possible to reverse network dysfunctions and contribute to ameliorating the onset and progression of neuropsychiatric diseases.

## 2. Molecular Characteristics of Neuroplastin

### 2.1. Structure and Expression of Neuroplastin

In humans and rodents, the small basigin gene family comprises three paralogs: basigin (*BSG*/*Bsg*; also designated CD147 or EMMPRIN), embigin (*EMB*/*Emb*), and neuroplastin (*NPTN*/*Nptn*) [33]. Neuroplastin isoforms were identified as glycoprotein components with molecular weights of 65 kDa (Np65) and 55 kDa (Np55) in isolated synaptic membranes from brain [34,35] (Table 1). The neuroplastin isoforms Np65 and Np55 are encoded by a single gene (*Nptn* in rodents, *NPTN* in humans) and result from alternative splicing of the mRNA [36]. Both isoforms are single-spanned transmembrane proteins belonging to the immunoglobulin (Ig) superfamily with two (Np55) and three (Np65) Ig domains, respectively. The intracellular carboxy-terminal tail of neuroplastin may also differ due to alternative splicing, resulting in variants that contain four additional amino acids Asp-Asp-Glu-Pro (DDEP) [36]. Glycosylation at several sites in the Ig2 and Ig3 domains results in a shift from the predicted molecular weight of 28 and 40 kDa to 55 and 65 kDa for the apparent molecular weight of the glycoproteins. Np55 is widely expressed with different glycosylated forms in many tissues such as the brain, liver, lung, and kidney [37], whereas Np65 is restricted to the brain, although it was also recently detected in cultured keratinocytes [38]. Both neuroplastin isoforms are expressed synaptically and extra-synaptically in excitatory and inhibitory neurons but have not been detected in glia [35,39,40]. In human and rodent brain, Np65 is strongly expressed in cortex, hippocampus, striatum, cerebellum, thalamus, and hypothalamus [35,39,40]. Np65 was also detected in both the inner and outer plexiform layers of the rat retina [41]. Np55 is expressed in all brain regions and is the major isoform in rodent cerebellum [42]. In the inner ear of the mouse, Np55 is expressed in the stereocilia of the outer hair cells, in the cell bodies of inner hair cells, and in spiral ganglia cells [43].

### 2.2. Interactions and Binding Partners of Neuroplastin in the Nervous System

#### 2.2.1. Neuroplastin Homophilic Binding and AMPA Receptor Subunit GluA1

**Figure 2:**
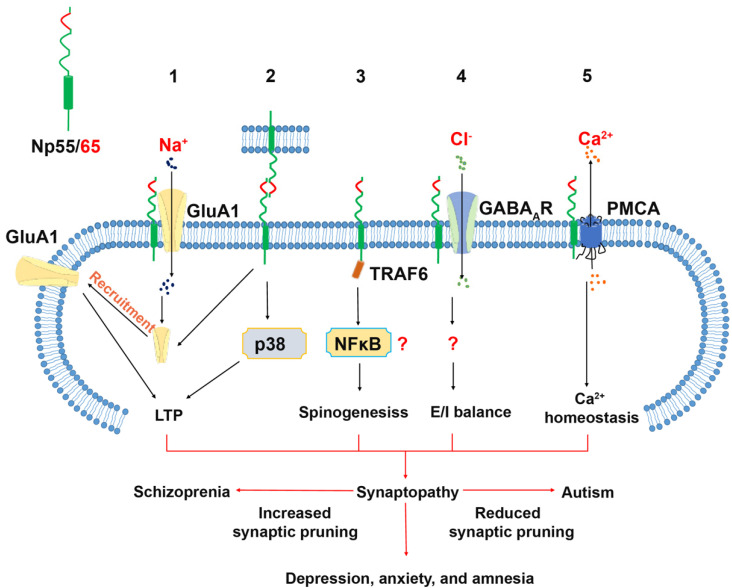
Neuroplastin binding proteins and their related cellular function in the central nervous system. **1.** The Ig1 domain of Np65 interacts with GluA1 supporting targeting of GluA1 to the plasma membrane and is required for the LTP maintenance. **2.** Neuroplastin trans-homophilic binding is involved in LTP maintenance. The binding motif resides in the Ig1 domain indicating that only Np65 can engage in this interaction. Homophilic binding of neuroplastin can be disrupted by the peptide "enplastin". Np55 and Np65 were proposed to bind homophilically in *cis*, however there are no explicit data supporting dimer formation. **3.** The intracellular tail of neuroplastin contains a TRAF6 binding motif, which is important for spinogenesis. **4.** Neuroplastin interacts with GABA_A_R. This interaction is critical for the balance of excitatory and inhibitory transmission. However, the binding domains are not identified. **5.** The transmembrane domain of neuroplastin is responsible for the interaction with the plasma membrane anchor domain TM10 of PMCA which regulates the extrusion of Ca^2+^ ions.

The adhesive capacity of the Np65-specific Ig1 domain to undergo trans-homophilic binding was first described using an aggregation assay of microspheres coated with neuroplastin-Fc chimeric proteins [39]. Later, crystallographic studies combined with Surface Plasmon Resonance confirmed that the Ig1 F-G loop of Np65 contains an adhesive binding site, and that this loop binds to the corresponding loop of an opposing recombinant Np65 with a *K*_D_ value of 0.52 ± 0.08 μM [46] (Figure 2). 

This interaction was blocked by enplastin, which is a dendromeric peptide derived from the Np65 trans-homophilic binding site itself [46]. The application of Np65-specific antibodies, able to block the aggregation of Np65-Np65, severely impaired the maintenance, but not the induction, of long-term potentiation (LTP) in CA1 neurons in rat hippocampal slices [39]. Furthermore, treatment with these antibodies resulted in a reduced cell surface expression of AMPA Receptor Subunit GluA1 and an increased phosphorylation of p38 MAPK [64]. These studies suggest that the blockade of potentially exciting trans-synaptic Np65-Np65 binding could result in electrophysiological deficits. Furthermore, incubation with the recombinant extracellular domain of Np65 caused loss of synaptic contacts in cultured hippocampal neurons [40]. Nevertheless, it remains to be confirmed whether in vivo trans-synaptic Np65-Np65 interactions exist and whether they confer structural stabilization to synaptic contacts. Alternatively, a recent study by Jiang et al. showed that the Ig1 domain of Np65 is specifically required for interaction with the extracellular N-terminal domain of GluA1. The absence of GluA1, or of its binding to Np65 as a receptor, resulted in impaired LTP maintenance [62]. GluA1 is critically important for LTP and is associated with various neurological diseases [65,66]. In GluA1-deficient mice, hippocampal LTP was absent without spatial reference memory deficits [67], but working memory deficits [68], schizophrenia-like behaviors [69,70] and increased locomotor activity, accompanied by reduced clearance of striatal dopamine, was displayed [69]. While GWAS identified GluA1 dysfunction as a risk factor for schizophrenia [12], molecular and pharmacological studies over the past decade have linked GluA1 to depression, anxiety, stress-related behavior, and Alzheimer‘s disease [71,72,73,74]. Interestingly, the levels of GluA1 expression and phosphorylation appear to be important for these neurological conditions [71,73,75,76]. In the future, it would be interesting to determine whether the phosphorylation state of GluA1 is modified by its interaction with Np65 or by the loss of neuroplastin.

#### 2.2.2. Neuroplastin and GABA_A_ Receptor (GABA_A_R)

GABA_A_ receptors were identified as potential binding partners of neuroplastin [47]. Indicative of a close interaction between Np65 and alpha1/alpha2 subunit-containing GABA_A_R (GABA_A_α1/α2) are fluorescence resonance energy transfer experiments performed in HEK cells, co-precipitation assays using rodent brain material, and co-localization analysis in cultured neurons and brain sections [47]. Loss of the association of neuroplastin and GABA_A_R may underlie the different GABA_A_α1/ GABA_A_α2 ratio in synapses and the altered inhibitory transmission in cultured neuroplastin-deficient hippocampal neurons [40], as well as in the hippocampus of neuroplastin-deficient mouse models [50]. The neuroplastin-GABA_A_ receptor association has not been determined on the atomic level, but the recently resolved structure of the GABA_A_ receptor [77] may contribute to resolving this issue.

The dysfunction of GABAergic transmission contributes to several neurological conditions, such as depression, anxiety, epilepsy (for a review, see: [78]), SZ [79], and ASD [80,81]. Based on the considerable combinatorial possibilities, a novel and more specific pharmacological intervention has been proposed as a potential advancement for clinical treatment over the use of nonselective GABA_A_ receptor agonists (for a review, see: [78,82]. Furthermore, accessory molecules that interact with GABA_A_ receptors may be new potential targets. Interestingly, neuroplastin expression has been linked to anxiety, depression, and 5-HT levels (see below, Depression and Anxiety Disorder). In neuroplastin-deficient mice, altered excitatory and inhibitory synaptic transmission was also observed [50]. The elucidation of the role of neuroplastin in the regulation and organization of the GABAergic system may contribute to a better understanding of the mechanisms underlying psychiatric disorders.

#### 2.2.3. Neuroplastin Binding to TRAF6

The tumor necrosis factor receptor-associated factor 6 (TRAF6), is an intracellular adaptor protein with E3 ligase activity that is largely known for its function in the activation and tolerance of immune cells, cell differentiation, and cancer [83,84,85,86]. TRAF6 is also involved in the regulation of programmed cell death that normally occurs during early development of the mesencephalon and diencephalon [87]. Furthermore, TRAF6 is proposed to play a role in Alzheimer’s disease (AD) and neuroinflammation [86]. TRAF binding sites have been identified in neuroplastin [38,60] (see below, association of neuroplastin to cancer). In particular, we showed that the binding of TRAF6 to its binding motif (RKRPDEVPD) within the C-terminal domain of neuroplastin promotes spinogenesis [60]. Genetic inactivation of *Nptn* or TRAF6-RNA interference strongly reduced the protrusion density of young hippocampal neurons, which could be rescued by the over-expression of Np55 or Np65. In mature neurons, TRAF6 does not co-localize with neuroplastin and does not promote spinogenesis, thus limiting the function of neuroplastin-TRAF6 interactions to the early neuronal spinogenetic phase [60]. Synapse malformation and alterations in synaptic density occurring during early neuronal development have been associated with schizophrenia [88,89]. Furthermore, altered TRAF6 mRNA levels were detected in hippocampus and striatum of SZ patients [90]. Therefore, it is tempting to speculate about a potential involvement of neuroplastin-TRAF6 interaction in the origin of schizophrenia (see below, associations of neuroplastin to schizophrenia).

#### 2.2.4. Neuroplastin Binding to Plasma Membrane Ca^2+^ ATPases (PMCA)

Recently, the expression of plasma membrane Ca^2+^ ATPases (PMCAs) was found to critically depend on neuroplastin in the mouse brain [35,50]. PMCAs are encoded by four distinct genes and expressed in numerous isoforms originating from alternative splicing [91]. PMCAs are essential for the extrusion of cytoplasmic Ca^2+^ to the extracellular side [92]. The loss of neuroplastin does not affect the transcription of PMCA genes [35], but in the absence of neuroplastin, the levels of PMCA proteins are reduced resulting in less Ca^2+^ extrusion and elevated intracellular Ca^2+^ levels with prolonged decay time to reach resting Ca^2+^ levels after stimulation [35,55,56]. Neuroplastin interacts directly with PMCAs forming functional complexes [35,55,56,57]. Gong et al. 2018 showed that the transmembrane domain of neuroplastin is responsible for binding to PMCAs. Cryogenic electron microscopy analysis of the neuroplastin–PMCA complex showed that the transmembrane domain of Np interacts with the 10th transmembrane domain and the 8th–9th transmembrane linker of PMCA, resulting in a conformational change required for the activity of PMCA and exposing the cytosolic Ca^2+^ binding site [57].

The function of the pairing of neuroplastin–PMCA must be regarded with respect to regulation of Ca^2+^ homeostasis, Ca^2+^ signal transduction, and synaptic activity, which are dysfunctional in neuropsychiatric diseases like ASD and SZ [93] and neurodegenerative diseases such as AD [94,95]. PMCA activity was found to be altered in AD human brain and was reduced by amyloid-β (Aβ) [96]. Furthermore, an interplay between PMCA with Aβ and tau protein was proposed [97]. Interestingly, decreased PMCA activity by Aβ or tau can be rescued by fostering the activity of the pump using endogenous regulatory proteins [98] or a synthetic phenothiazine [99]. In addition, numerous PMCA mutations are associated with human diseases and impairments, like deafness and ataxia (for review see: [100]). Interestingly, the genetically driven ablation of *Nptn* results in decreased PMCA levels and deafness in mice ([43]; see below, Section 3.5.1 Deafness).

## 3. Neuroplastin in Neurological Diseases

A particular syndrome has not yet been attributed to neuroplastin malfunction. However, the observed functions in mouse models and the expression, structure, and interaction partners of neuroplastin, indicate that the impairment of neuroplastin function in humans may result in deleterious consequences for the nervous system. Here, we will review the evidence for the contribution of neuroplastin to neurological pathologies.

### 3.1. Schizophrenia (SZ) and Autism Spectrum Disorder (ASD)

SZ and ASD manifest as distinct neurodevelopmental diseases. ASD frequently presents in childhood, whereas SZ manifests later in young adults. For both disorders, a heritable genetic contribution was observed, but explicit monogenetic causes have not been identified. Furthermore, a significant association between ASD and SZ was detected [101]. Strikingly, many gene loci related to synaptic function were identified as contributing to both SZ and ASD, suggesting that pathological malfunctions of synapses or synaptopathies may be causal (for review see: [102]). In addition, these two diseases frequently co-occur with attention deficit hyperactivity (ADHD) and bipolar disorder (BD); this is likely resulting from a developmental synaptopathy [103,104]. Brain images from ASD children have shown increased brain size and weight [105] affecting axons and synaptic density [106], which indicate an acceleration of brain development and more synaptic connections. A lack of adolescent synaptic pruning was observed in ASD patients [107], which may account for the dysfunction of brain circuits in ASD [108].

Unlike ASD, which shows an increase in brain growth in all brain regions except occipital grey matter [109], loss of grey matter in SZ was observed [110]. Excessive synaptic pruning in prefrontal cortical synapses was found in SZ neuropathology [111], which indicated reduced synapses and further impairments of the brain circuitry and cognitive functions [112,113].

#### 3.1.1. Neuroplastin Relation to Schizophrenia

In rat models displaying schizophrenia-like symptoms after injection of the two different psychostimulants methamphetamine (MAP) or phencyclidine (PCP), neuroplastin was significantly up-regulated [114]. MAP is a dopamine transporter inhibitor that causes a positive symptom, clinically similar to paranoid schizophrenia in an acute phase [115]. PCP is an NMDA receptor antagonist, which induces both negative and positive schizophrenia-like symptomatology [116]. Subsequent genetic studies of patients with schizophrenia identified three single-nucleotide polymorphisms (SNPs) in the 5′-upstream region of *NPTN* that were strongly correlated to schizophrenia [44].

Pre-pulse inhibition (PPI) of the startle response is often considered as a characteristic in the diagnosis of schizophrenia [117]. In *Nptn*-deficient mice, PPI is severely impaired [50], although this could simply result from the profound hearing deficit of these mice [43], rather than processing deficits. Nevertheless, the significant reduction in paired-pulse facilitation in the auditory cortex of *Nptn*-deficient mice suggests altered cortical synaptic transmission [43]. In addition, the PPI deficit in heterozygous *Nptn*-deficient mice [50] points to potential central alterations as contributors to the phenotype. As detailed above, the neuroplastin interaction partners AMPA receptor subunit GluA1 and TRAF6 have also been associated with schizophrenia.

#### 3.1.2. Autism Spectrum Disorder (ASD)

Some patients suffering from the heterogeneous 15q24 microdeletion syndrome display ASD and attention deficit hyperactivity disorder (ADHD), in addition to various other deficits [118,119]. *NPTN* is located at cytogenetic band 15q24.1 and it is deleted or duplicated in some 15q24 microdeletion syndrome patients [118,119]. Furthermore, PMCA2 was identified by GWAS studies to be associated with ASD [120]. A study which included 717 children associated the cortical morphology, such as cortical thickness and surface area, with autistic traits [121]. Interestingly, a single-nucleotide polymorphism in *NPTN* was found to be associated with cortical thickness [49]. Furthermore, the paths to ASDs may involve unbalanced excitatory–inhibitory synaptic transmission and abnormal synaptogenesis [122]. Several studies have observed an imbalance of excitatory-inhibitory transmission and altered synaptogenesis in different *Nptn*-deficient mice [40,51,59,60,123]. In addition, neuroplastin-deficient mice displayed altered social interactions avoiding unfamiliar mice [50]. In conclusion, genetic association studies suggest a link of neuroplastin to autism spectrum disorder, but the role of *NPTN* in ASD still needs to be specifically addressed. It remains to be seen whether a direct malfunction or loss of neuroplastin, rather than an indirect effect, e.g., via PMCAs, contributes to ASD.

### 3.2. Depression and Anxiety Disorder

Depression and anxiety are the most common mental disorders in society today, and both frequently co-occur in patients [124,125]. Etiological factors related to depression and anxiety disorder could be linked to childhood trauma, environmental adversity, as well as stressful life events [126]. Furthermore, several genes have been associated with depression and anxiety, among them *5-HTT*, *NPSR1*, and *RGS2* [126]. Genetic inactivation of *Nptn* results in elevated corticosterone levels and increased depressive-like behavior, but reduced anxiety-related behaviors in mice [50]. Mice that lacked only Np65 displayed the opposite phenotype, with reduced depressive-like behavior and increased anxiety [59]. In addition, the neuroplastin binding partners GluA1 and GABA_A_ receptor are associated with anxiety disorder and depression (see Section 2.2.1 and Section 2.2.2).

### 3.3. Alzheimer’s (AD) Disease

**Figure 3:**
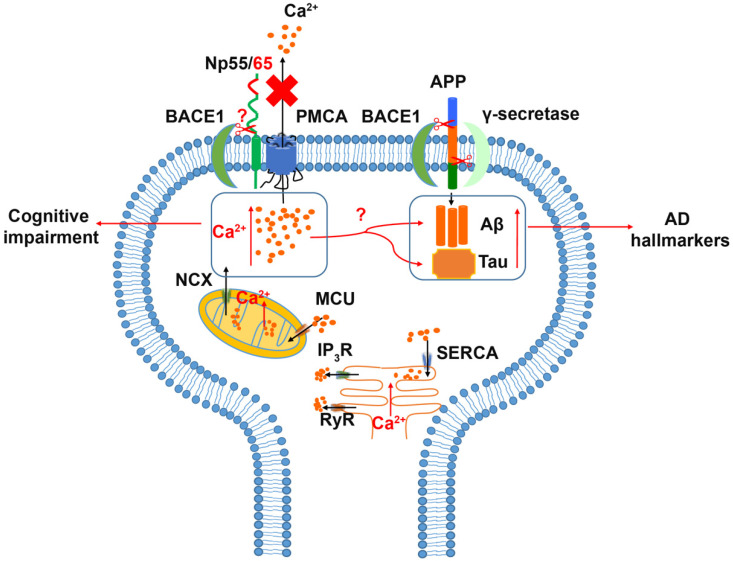
Relation of Neuroplastin to Alzheimer’s disease. The Amyloid Precursor Protein (APP) is cleaved aberrantly by β-secretase (BACE1) and γ-secretase resulting in Aβ. Aβ and tau are considered hallmarks of Alzheimer’s disease. Increased intracellular Ca^2+^ concentrations are associated with cognitive impairment and increases in Aβ and tau. Intracellular Ca^2+^ can be deposited into or released from mitochondria and ER as intracellular calcium stores. Energy driven extrusion of Ca^2+^ is mediated by PMCAs. In the absence of neuroplastin, PMCA levels are reduced and intracellular Ca^2+^ is increased. The hypothetical cleavage of neuroplastin by BACE1 may result in loss of PMCAs and elevated Ca2+ levels.

An alteration of neuroplastin expression in AD patients was reported recently [58]. In the early phase of confirmed AD neuropathology, neuroplastin was significantly upregulated in the hippocampus (dentate gyrus, CA2/3 region, and subiculum) without changes in neuron number or tissue volume. Interestingly, patients experiencing a longer duration of AD disease (5–7 years) showed a decreased expression level of neuroplastin compared to patients with a short duration AD (≤4 years), which may indicate a role of neuroplastin in the early phase of AD. The analysis of neuropathological amyloid plaques and neurofibrillary tangles (NFT) showed a negative correlation between neuroplastin expression level and the number of amyloid plaques in the CA1 area and a weak negative correlation between neuroplastin and NFT in CA1, CA2/3, and subiculum. In human brain, both *NPTN* and *PMCA*s exhibit similar expression patterns at the transcriptomic level [127]. In comparison to the aging brain, the expression and activity of PMCA in AD patients were reduced, and the AD hallmarks tau and Aβ showed a negative impact on PMCA activity, which may indicate an altered Ca^2+^ homeostasis in the AD brain [96,128,129]. Alternatively, Ca^2+^ dys-homeostasis could promote the accumulation of Aβ and phosphorylated tau protein, which result in the neuropathy and brain function deficits in AD patients [128,129,130]. Aβ is produced by the β- and γ-secretase cleavages of the amyloid precursor protein (APP) [131]. The principal β-secretase for generation of Aβ in vivo is the β-site APP cleaving enzyme 1 (BACE1) [131,132]. The use of BACE1-specific inhibitors has been proposed as a potential intervention in AD [133]; however, this approach must be regarded carefully, as BACE1 cleaves numerous substrates. Interestingly, both neuroplastin and basigin were identified as potential BACE1 substrates [134,135]. Although further studies on the cleavage of Np by BACE1 were not conducted, an attractive hypothesis is that increased neuroplastin cleavage by BACE1 could result in the cognitive deficits observed in AD (Figure 3).

### 3.4. Cognition, Antero- and Retrograde Amnesia

A large-scale genetic association study in adolescents associated *NPTN* with cortical thickness and intellectual ability [49], suggesting a role for NPTN in cognition and learning and memory. Furthermore, *NPTN* variants were recently related to creativity [136].

In recent years, we have addressed the role of neuroplastin in learning and memory using several mouse mutants. When neuroplastin was missing from only glutamatergic neurons, achieved using an *Emx1*-promoter driven Cre-recombinase, associative learning was slightly improved. However, the continuity of task execution was affected, suggesting altered striatum-dependent decision making [35]. The complete loss of neuroplastin expression resulted in a complex phenotype, which included the inability to learn associative tasks [50]. The comparison of *Nptn*-ablation in glutamatergic versus all neurons suggests a particular role of neuroplastin, expressed by gabaergic interneurons for associative learning. When neuroplastin expression was specifically ablated in all types of neurons after a normal development, again, the anterograde memory was not formed for associative tasks [50]. Furthermore, when the associative tasks were first acquired perfectly before neuroplastin ablation, the induced loss of neuroplastin resulted in specific retrograde amnesia for these associative memories but not for spatial memories [50]. Interestingly, the β-blocker propranolol has retrograde amnestic side effects. Therefore, propranolol is applied as off-label use for the treatment of intrusive thoughts associated with post-traumatic stress disorder (PTSD) (for a review, see: [137]). Propranolol also acts as an inhibitor of the PMCAs [138], and thus its amnestic effects may be related to PMCA inhibition. In vivo, the absence of neuroplastin leads to dramatically reduced PMCA levels [35,50], suggesting that the neuroplastin-PMCA assembly may be critical for associative learning and memory. If this hypothesis can be substantiated, it may provide an opportunity to address PTSD and other psychiatric conditions involving intrusive thoughts.

### 3.5. Other Diseases Related to Neuroplastin

#### 3.5.1. Deafness

Deafness resulting from the loss of neuroplastin function has been studied using *Nptn*-deficient and neuroplastin mutant mice [43,51,52]. It was proposed that Np55 expression by outer hair cells is required for cochlear amplification [52], and Carrott et al. [51] proposed Np65-driven synaptogenesis by inner hair cells as necessary for hearing. Recently, we showed that neuroplastin expression is essential for hearing during the development of the hearing system, and also for the maintenance of hearing capabilities in adults throughout their life [43]. Neuroplastin is required for PMCA2 targeting and Ca^2+^ extrusion in cochlear hair cells [43]. Interestingly, PMCA2 loss of function mutations result in deafness in mice and human [139], verifying that the interaction of neuroplastin with PMCA is decisive for Ca^2+^ extrusion.

#### 3.5.2. Cancer

The first evidence linking *NPTN* to cancer came from a bioinformatic analysis showing that *NPTN* was one of 166 genes with an altered expression in colorectal adenomatous polyps [140]. In a study screening for potential tumor antigens from breast cancer patients, neuroplastin was identified and showed strongly increased immunoreactivity in invasive carcinoma tissues [141]. Moreover, over-expression of neuroplastin in a breast-cancer-derived cell line strongly increased tumor growth and angiogenesis, as well as the production of vascular endothelia growth factor (VEGF), suggesting an angiogenic mechanism regulated by VEGF in the aberrant neuroplastin-expressing tumors [141]. Furthermore, the role of neuroplastin and its interaction with S100A8/A9, resulting in the activation of a cascade for lung cancer disseminative progression and aggressive development, has been proposed [142,143].

#### 3.5.3. Various Diseases

Not surprisingly, the widespread expression of neuroplastin in nearly all organs may result in the discovery of further pathological conditions influenced by neuroplastin, e.g., within the immune system [55] or in heart disease [144].

## 4. Future Research Directions

The multiple binding partners place neuroplastin centrally in the interwoven processes of (a), synapse formation and synaptic plasticity, and (b), intracellular calcium signaling. While developmental dysfunctions of neuroplastin-mediated synaptic processes may be more related to neuropsychiatric diseases, neurodegenerative diseases may instead involve neuroplastin-related alterations in synaptic calcium extrusion. However, both aspects must not be mutually exclusive. Therefore, the association of neuroplastin, PMCAs, and AMPA receptors in synaptic assemblies will be a topic of further investigation. Furthermore, the potential cleavage of neuroplastin by BACE1 or other proteases, and its successive cognitive impairment and neurodegeneration, should be addressed. It is likely that neuroplastin variants will be identified as causal for specific human disease syndromes, possibly not only affecting the nervous system. Future research will address by which mechanisms neuroplastin influences learning and memory. Of particular interest is the loss of associative memories after neuroplastin ablation. The possibility to elicit retrograde amnesia in a controlled manner in a mouse model allows for the study of underlying mechanisms, and can increase the understanding of the molecular and circuit processes of memory. This retrograde amnesia model opens experimental means of developing treatment approaches for posttraumatic stress disorder, traumatic experiences, and intrusive thoughts. In particular, the analysis of neuronal subsets and the role of neuroplastin expression in gabaergic interneurons may reveal decisive circuits for associative memories.

## 5. Conclusions and Perspectives

The study of neuroplastin in recent decades has elucidated a complex and interwoven network of binding partners. Neuroplastin is evolving as an important molecule, with essential functions in the nervous system for optimal synapse formation, synaptic plasticity, and learning and memory. Accordingly, the functions of neuroplastin can now be related to neuropathological conditions. Expression in most organs implies a critical function of neuroplastin, which may be affected in other diseases, such as cancer or heart disease. In particular, the recent identification of neuroplastin as a decisive component of plasma membrane Ca^2+^ ATPase complexes has opened new perspectives for the mechanistic understanding of learning and memory processes. The unique animal model for induction of retrograde amnesia may help to understand mechanistically memory loss, and might provide useful insights to develop further strategies for the treatment of PTSD.

## Figures and Tables

**Table 1:** History of discoveries in neuroplastin research.

Year	Main Incident	References
1988	Neuroplastins are first described as glycoproteins in synaptic membranes	[34]
1997	Neuroplastins are Ig superfamily members with similarity to basigin	[36]
2000	Np65 is involved in LTP	[39]
2001	*Nptn* expression in rat retina	[41]
2006	Np65 activates p38 MAPK and regulates surface GluR1 and LTP	[37]
2007	*NPTN* is associated with developmental delay and schizophrenia	[44]
2010	Np55 interacts with FGFR promoting neurite outgrowth	[45]
2011	Extracellular Np65 binding to Np65 regulates neuritogenesis	[46]
2012	Np65 co-localizes with GABA_A_ receptor	[47]
2013	Neuroplastin might chaperone MCT2	[48]
2014	Np65 regulates the number and function of excitatory and inhibitory synapses	[40]
2015	*NPTN* is associated with cortical thickness and intellectual ability in adolescents	[49]
2016	Retrograde amnesia of associative memories and PMCA loss after inducible *Nptn* deletionNeuroplastin is required for learning and memory	[50]
2016	*Nptn* is identified as a deafness gene	[51,52]
2016	Neuroplastin-kr8 complex in apoptotic phosphatidylserine exposure	[53]
2016	Np65 as receptor for S100A8/9A signaling via GRB2 and TRAF2	[38]
2017	Np65 KO mice are more susceptible to ischemic brain injury	[54]
2017	Neuroplastin elimination in glutamatergic neurons causes PMCA loss and behavioral alterations in mice	[35]
2017	Neuroplastin–PMCA complexes	[55,56]
2018	Cryo-EM structure of Neuroplastin–PMCA1 complex	[57]
2019	Neuroplastin expression in AD	[58]
2019	Np65 KO mice exhibit anxiety and depression-like behavior	[59]
2020	Neuroplastin interacts with TRAF6 to promote spinogenesis	[60]
2020	Neuroplastin interacts with MANF to regulate inflammatory responses	[61]
2021	Neuroplastin–GluA1 interaction mediates LTP	[62]
2021	Neuroplastin is essential for hearing and hair cell PMCA expression	[43]
2021	Neuroplastin is related to aging and chronic stress	[63]

## Data Availability

N/A.
